# Editorial: Challenges and advances in carcinomatous meningitis treatment

**DOI:** 10.3389/fonc.2025.1672755

**Published:** 2025-08-26

**Authors:** Maciej M. Mrugala, Katherine B. Peters

**Affiliations:** ^1^ Mayo Clinic Arizona, Scottsdale, AZ, United States; ^2^ Duke Cancer Institute, Durham, NC, United States

**Keywords:** cerebro-spinal fluid (CSF), leptomeningeal disease, liquid biopsy, intrathecal chemotherapy, circulating tumor DNA (ctDNA), circulating tumor cells (CTCs)

Leptomeningeal metastasis (LM), the dissemination of malignant cells into the cerebrospinal fluid (CSF) and leptomeninges, represents one of the most aggressive and therapeutically challenging complications of advanced solid tumors (1). Historically associated with dismal prognosis and limited treatment options, LM has remained a final frontier in oncologic care, particularly in patients with non-small cell lung cancer (NSCLC) and breast cancer. However, a wave of new research, including four recently published studies that we are sharing with you in this special edition of *Frontiers in Oncology* is reshaping our understanding of LM pathobiology, diagnostics, and treatment. Collectively, these studies point to a future in which CSF-directed therapies and molecular profiling are central pillars of LM management, offering hope where previously there was very little.

## Intrathecal pemetrexed combined with systemic therapy in NSCLC: a promising strategy

The retrospective study from the First Affiliated Hospital of Gannan Medical College highlights the growing role of intrathecal (IT) chemotherapy in treating LM from NSCLC (Zhong et al.). Thirty-one patients received IT pemetrexed in conjunction with systemic therapy, achieving a striking 87.1% intracranial disease control rate and a median intracranial progression-free survival of 9 months. This observation is a marked improvement over historical controls, where LM patients often survive less than six months following diagnosis.

The tolerability profile was also encouraging. Most adverse events were low-grade, and only four patients experienced grade 3 toxicity. Importantly, survival was significantly better in patients with good performance status (ECOG ≤1) and those receiving systemic bevacizumab, suggesting possible synergy between anti-angiogenic therapy and IT chemotherapy.

This study underscores the value of bypassing the blood-brain barrier (BBB) to deliver cytotoxic agents directly into the cerebrospinal fluid (CSF). While pemetrexed is well established systemically in NSCLC, its intrathecal use is uncommon and demonstrates that repurposing familiar agents for CNS disease may provide meaningful benefit (2). However, as a retrospective single-center analysis with a small sample size, the findings require validation in prospective, controlled trials.

## A rare case of BRAF non-V600E LM in NSCLC: expanding the molecular horizon

A complementary case report by Li et al. presents a unique clinical narrative: a 63-year-old woman with LM from BRAF non-V600E mutant NSCLC experienced 24 months of survival following treatment with intrathecal chemotherapy with pemetrexed and trametinib, an orally available inhibitor of MEK1 and MEK2. This outcome is extraordinary in the context of EGFR/ALK-wild-type NSCLC, where median survival in LM typically ranges from 2 to 4 months.

The case report is important not only because of its duration of disease control but also because it demonstrates the clinical utility of CSF-based molecular profiling. While the primary tumor lacked actionable targets, CSF next-generation sequencing (NGS) revealed multiple non-V600E BRAF mutations and CDKN2A loss—mutations rarely addressed in standard NSCLC care. This critical result allowed clinicians to pursue a rational off-label treatment with trametinib, which appeared to stabilize the disease for nearly a year.

This patient’s course also highlights the inadequacies of traditional diagnostic tools. MRI failed to identify LM definitively; diagnosis relied on CSF cytology and pressure measurement. This finding reiterates the urgent need for improved CSF diagnostics—not just to confirm LM, but to inform treatment decisions based on evolving tumor genomics.

## CSF liquid biopsy: the diagnostic and prognostic frontier

In a comprehensive review, Pentsova outlines how CSF-based liquid biopsies—specifically circulating tumor cells (CSF-CTCs) and circulating tumor DNA (CSF-ctDNA)—are transforming the diagnosis and management of CNS metastases. Traditional tools, such as MRI and CSF cytology, suffer from low sensitivity and specificity. CSF liquid biopsy offers a minimally invasive and biologically informative alternative.

Studies show that CSF-CTC counts (≥3 cells/3ml) can identify LM with high diagnostic accuracy (93% sensitivity, 95% specificity). Meanwhile, CSF-ctDNA can reveal tumor mutations, resistance mechanisms, and clonal divergence from primary tumors. In CNS metastases, plasma ctDNA is often undetectable due to the BBB, making CSF a uniquely valuable biospecimen.

Moreover, changes in CSF-CTC burden and ctDNA variant allele frequencies may serve as real-time biomarkers of disease progression and treatment response, which are critical capabilities in a disease that evolves rapidly and is difficult to monitor. As CSF liquid biopsy technologies mature, they promise to enhance not only diagnosis but also personalized treatment planning and clinical trial eligibility.

## HER2 “flip” in breast cancer LMD: challenging therapeutic assumptions

Perhaps the most paradigm-shifting insights come from a retrospective analysis of 26 patients with breast cancer LM by Kumthekar et al., revealing frequent discordance between HER2 status in the primary tumor and in CSF tumor cells. Using the CNSide™ platform, HER2 amplification was identified in 35% of patients whose primary tumors were initially classified as HER2-negative. This phenomenon termed “HER2 flip”, thus highlights the dynamic and compartmentalized nature of tumor evolution, especially in the CNS.

Serial testing in 14 patients showed that HER2 status can change over time, even during treatment. These fluctuations had direct therapeutic consequences, prompting the initiation or cessation of HER2-targeted therapies, such as intrathecal trastuzumab. In patients with HER2-positive CSF tumor cells, treatment led to tumor burden reduction and clinical stabilization.

Moreover, CNSide demonstrated significantly higher sensitivity than traditional cytology, detecting tumor cells in 100% of initial CSF samples compared to just 65% with cytology. Beyond detection, CNSide also quantifies tumor burden and enables HER2 biomarker analysis, offering a comprehensive diagnostic snapshot that cytology cannot provide.

This study makes a strong case for routine CSF biomarker testing in LM, regardless of the primary tumor’s profile. It also challenges the longstanding notion that metastatic disease mirrors its origin in actionable features. As a result, it opens new therapeutic doors for patients previously considered ineligible for targeted treatment.

## A common thread: precision medicine, driven by CSF

What unites these four studies is a shared vision of CSF as more than a diagnostic fluid—it is a window into tumor biology, evolution, and treatment response. In both lung and breast cancer, molecular profiling of CSF is revealing actionable mutations, resistance patterns, and tumor heterogeneity that are not captured by plasma or primary tissue testing. Whether guiding the use of MEK inhibitors in BRAF-mutant NSCLC, determining eligibility for HER2-targeted therapies, or monitoring treatment response, CSF analysis is emerging as the cornerstone of CNS-directed precision oncology.

Furthermore, the growing body of evidence supports a more aggressive, multimodal treatment paradigm for LM, integrating intrathecal chemotherapy, targeted agents, and serial molecular surveillance. This approach moves beyond the nihilism that often surrounds LM and offers a roadmap for extending survival and improving quality of life.

## Looking ahead: from retrospective insight to prospective impact

While these findings are compelling, the path forward requires rigorous validation. Most data are retrospective or anecdotal, and sample sizes remain small. Prospective, multicenter trials with standardized protocols are necessary to confirm efficacy, assess long-term toxicity, and determine the optimal treatment sequences.

Additionally, broader access to CSF-based diagnostics, including NGS, ctDNA, and CTC platforms, must be prioritized. Currently, such technologies are restricted to specialized centers and remain cost-prohibitive for many institutions. Investment in infrastructure, reimbursement, and education will be critical to democratizing these advances.

## Conclusion

Together, these studies chart a hopeful trajectory in the management of leptomeningeal metastases. They call for a redefinition of what is possible whether it is clinically, diagnostically, and biologically, in a disease long considered untreatable. By embracing CSF as a source of real-time, compartment-specific tumor intelligence and integrating targeted therapies that reach the CNS, oncology is finally turning the tide in LM. The future of LM care is no longer defined solely by palliation; it is being reshaped by precision.
